# Acclimation during space flight: effects on human emotion

**DOI:** 10.1186/s40779-016-0084-3

**Published:** 2016-04-29

**Authors:** Qing Liu, Ren-Lai Zhou, Xin Zhao, Xiao-Ping Chen, Shan-Guang Chen

**Affiliations:** Department of Psychology, School of Social and Behavioral Sciences, Nanjing University, Nanjing, 210023 China; School of Psychology, Beijing Normal University, Beijing, 100875 China; Research Center of Emotion Regulation, Beijing Normal University, Beijing, 100875 China; School of Psychology, Northwest Normal University, Lanzhou, 730000 China; China Astronaut Research and Training Center, Beijing, 100094 China

**Keywords:** Space flight, Weightlessness, Emotion, Physiology

## Abstract

Recently, studies on the extent to which spaceflight affects the psychology of individuals has received attention. In order to reveal the mental challenges that humans face in space, we need practical viewpoints to integrate the psychological effects, behavior, performance and the environment itself for space exploration. The present review discusses the individual variables related to space psychology and manned spaceflight, in addition to their growing trends. These items include patterns of emotional changes in extreme environments and the approaches to evaluating emotions. Moreover, the review concludes with suggested future research on emotion during spaceflight and its analogs. These data and information are needed to plan for the exploration of the Moon and Mars, along with contributions to the construction of the international space station (ISS) and astronaut training.

## Background

### Emotion and its measurement

The exploration of emotion has been the focus of psychologists for many years. Emotion has played an important role in human evolution and has had an indispensable effect on human life. Researchers have thought that emotion is a comprehensive process that changes over time. Emotion has multiple patterns of manifestations that include subjective experience, facial expression and both central and peripheral nervous system reactions. Other manifestations include changes in cognition, information processing and behavior. All these manifestations have complicated connections and interactions. Therefore, the measurement of emotion should be conducted within the interactions of multiple factors [1].

Subjective reports use emotional rating scales and questionnaires that examine other related content to allow participants to indicate their emotional reactions during recent periods. It is the simplest and most practical way to measure a study participant’s emotions [2]. The autonomic nervous system (ANS) is a branch of the peripheral nervous system that is responsible for the regulation of visceral functions. The ANS is composed of the sympathetic nervous system (SNS) and the parasympathetic nervous system (PNS), which typically have opposing excitatory and inhibitory effects. The activity of the ANA is not only related to emotional reactions but also plays a major role in many related physical functions, such as digestion, because of its autonomic character. It is unknown whether the measurement of ANA activity during emotional reactions reflects ANS activity itself or its auxiliary functions related to emotional states [3]. The measurement of electroencephalogram (EEG) activity revealed that frontal asymmetry was related to emotional valence. The study of Sutton and Davidson [4] showed that activation that included a left frontal asymmetry was associated with approach motivation, whereas activation that included a right frontal asymmetry was associated with avoidance motivation. Brain imaging studies have demonstrated that the complicated reactions of emotional states occurred through the connectivity of multiple brain areas and not the activity of a single brain area [5]. Many studies have shown that the emotional states that are connected to approach or avoidance motivation can be functional localized in brain connectivity [6].

Any single method for measuring emotion has its limitations because of the complexity of emotions. Therefore, to include the complicated changes of emotion, researchers have proposed the combination of different measurements of emotion, such as observer ratings, facial measurement, ANS activity, brain activity and voice measurement. Using this approach, we can eventually understand emotional changes. To investigate emotion in extreme environment, such as spaceflight and ground-based simulation, we have used subjective emotional reports [Beck anxiety inventory (BAI), Beck depression inventory (BDI) and the positive affect and negative affect scale (PANAS)] [7–9], objective physical recordings [galvanic skin response (GSR), heart rate (HR) and heart rate variability (HRV)] [10–12] and brain activity (EEG asymmetry) [13, 14]. By using multiple measurements, we can fully demonstrate the effects of spaceflight and its effects on emotions of individuals.

## The extreme environment of spaceflight and its character

Spaceflight puts humans into an extreme environment in which they cannot naturally adapt, and as a result, they must experience complex physical and psychological adaptation processes to survive. These types of environments, such as outer space, are called extreme environments [15, 16]. The authors of the present review participated in the Chinese manned spaceflight ‘973’ national major project named ‘the study of variation discipline and mechanism of the operation performance for crew members under long-duration spaceflight’. Therefore, we will discuss and compare different research items and demonstrate their interactions and relationships, as well as the working variables and possible countermeasures, under a common theoretical and practical consensus. Here, we provide all references under each item, summaries and prospects for future research.

Spaceflight has unique physical characteristics. Among them, the most outstanding is the lack of gravity. Fundamental changes between spaceflight and life on Earth affect almost all systems of the human body. At the same time, cognition and psychomotor functions are also influenced by microgravity. The head-down bed rest (HDBR) protocol used on Earth has been shown to be an effective tool for manipulating the effects of weightlessness on individual physiology [17, 18]. A second unique physical characteristic of spaceflight that differs from life on Earth is the change in the body’s natural circadian rhythms. From the perspective of physiology and psychology, microgravity and variations in circadian rhythms are two factors that require complex preparation for human spaceflight, and these factors are the most important areas of focus for human space exploration [19]. The changes in circadian rhythm day-night cycles provide the largest disruption to an individual’s sleep cycle. Therefore, a sleep deprivation experiment was conducted on Earth to mimic the effects of changes that occur between day and night on individual physiology [20].

The second variable for spaceflight is the stress that humans experience while living in an extreme environment, which results from factors of spaceflight such as habitability, mental functions and interpersonal relationships [21]. These factors are not unique to spaceflight, as they are common characteristics of limited and isolated environments. However, these traits are important for living and working in a spaceflight environment, especially for long-duration spaceflight. Similar to the unique physical characteristics of spaceflight (microgravity and circadian rhythm variation), the stress factors experienced in space were also issues the astronauts and their crew has to adapt to because maintain a high level of efficacy for crew members is vital to the survival and success of the spaceflight [22]. This is why an equivalent environment on the ground could be used to investigate the effects of the space environment on an individual’s physiology and psychology because microgravity and circadian rhythm variation can be used in such an environment and could induce human stress reactions.

There are at least two factors that could weaken an astronaut’s cognition and psychomotor functions. The first factor relates to the direct and specific effects of microgravity on brain functioning, especially the effects on the vestibular and motor systems. The second factor relates to nonspecific and stress effects, such as cumulative insomnia, workload or the physical and emotional stress caused by living and working conditions in the space capsule. Unlike the effects of microgravity, nonspecific effects do not affect psychological or physiological functions directly but instead influence patterns of physical activity, which subsequently affect the process functions. Therefore, when considering the effects of nonspecific factors on an individual’s stress reactions, we should also consider the effects of physiological variation as the moderating variable. Therefore, when discussing testing measures, we should combine subjective and objective methods to fully demonstrate the effects of the nonspecific factors [7–10]. The nonspecific stress states include a decrease in alertness, fatigue, a strong workload and emotional stress caused by interpersonal relationships or the condition of limitation and isolation itself. Another problem that should not be neglected is the individual differences in positive coping strategies in stressful conditions.

## Effects of spaceflight and analog on emotion

Thus far, experimental findings have identified one specific stress factor, which is microgravity. Microgravity can impair an individual’s cognition and psychomotor functions during the primary phase of spaceflight. However, most effects are quite specific and may not fully demonstrate the possible changes in an astronaut’s performance and operation in a space environment. One problem is that previous studies that focused on potential stress-related damage did not consider the effects of other nonspecific factors, such as workload, sleep disturbance and emotional tension. Therefore, the present review is based on the effects of specific and nonspecific factors on an individual’s emotional and mood changes in the space environment. We chose the effect of time simulation methods and interpersonal interactions as our themes to demonstrate the possible changes in subjective reports, objective physiology, reactivity and neural activity. The main purpose of this review is to illustrate the variations in emotions and mood during spaceflight tasks that are related to variables with specific and nonspecific effects.

Because spaceflight is a complex project with a high cost and high risk and requires large amount of manpower, some research into spaceflight is still in the primary stages. Although experiments conducted on Earth are more economic and safe compared to the environment of space, there are more control variable in the analogs and the operation items can reflect space, there are more control variables available in the analogs and the operation items can reflect those of spaceflight. In considering the variables of microgravity and circadian rhythms, we chose the analogs of −6° (15 days and 45 days) and the conditions of 72 h of social isolation and sleep deprivation. In addition, we observed emotional changes during 13 days of spaceflight. From the perspective of integrating the ground analog and real spaceflight, we investigated emotional changes in extreme environments. This could provide empirical evidence for emotional changes during long-duration spaceflight and in the international space station (ISS).

However, considering the time course is more challenging. In other words, to demonstrate the possible variations in performance over time, several performance evaluations would generally be conducted in one spaceflight.

Previous studies have suggested the short-duration spaceflight would not affect an astronaut’s performance or psychology because most people could beat the stress factors in short-duration spaceflight. However, long-duration spaceflight may have more complex effects on astronauts because the astronaut’s ability to cope with stress is limited and there will be new stress factors in long-duration spaceflight. Therefore, we detailed the issues of time accumulation during spaceflight and will conduct our experiment through dynamic tracking and static observation. We carried out short-duration and long-duration HDBR using dynamic tracking to measure the emotional changes before, during and after HDBR. In addition, considering the effects of circadian rhythms on the individual, we also began the 72-hour sleep deprivation experiment under social isolation, which compares emotional changes before and after sleep deprivation from the perspective of static observation.

It is well known that extreme environments have an effect on interpersonal interactions. As for the methodology used to investigate the effects of an extreme environment on an individual, we should consider the individualization analysis method. We will need to observe emotional changes at the individual level rather than the group (mean) level because group observations may obscure variations in important results.

Such variation would result from large inter-individual differences, which relates to different methods for measuring emotions and different profiles of emotional reactivity. For the former, we would consider using subjective reports (PANAS) and objective (GSR, HR, HRV) or a neural index (frontal EEG asymmetry) of variations to conduct the study. For the latter, we would observe spontaneous emotional responses and on-going emotional regulation processes to choose an index for observing emotional changes.

According to the above three factors, we identified the related papers and reviewed the main findings. A study regarding emotion in the environment of space proposed a four-stage model emotional change [23]. Specifically, the first stage was the psychological discomfort stage, which was caused by physical discomfort (e.g., headache, back pain). The second stage occurred approximately six weeks after spaceflight, when the astronauts had adapted psychologically and physically but were not yet affected by the isolation, defined and single life. The third stage was the most important stage for emotional changes, which occurred from the sixth week to the twelfth week. During this stage, because of the effects of isolation, defined and single life, the astronauts became less stable emotionally, showed irritability during their performance, had lower energy levels and were fond of stimulating music. The last stage occurred at the end of the spaceflight, where the astronauts showed a feeling of euphoria. Bechtel and Berning [24] also emphasized this third stage of spaceflight, regardless of whether the spaceflight was short (a few weeks) or long (a few months or years). They came up with the “three-quarter phenomenon”, indicating that emotion and personal issues increased significantly in the third stage of spaceflight.

The adaptive stage model (three-stage model) propsed by Rohrer considers the effects of isolation and confinement in extreme environments on a crew member’s emotions, performance and interpersonal relationships. In the first stage, the individual feels more anxiety and nervousness. In the second stage, the individual feels depressed because of a monotonous and boring life. In the third stage, the individual shows obvious hostility [25]. Style and his colleagues found that HDBR may be a useful method for inducing low back pain, mood changes, and changes in self-reported activity levels in bed rest studies [26]. Ishizaki et al. [27] considered that the implementation of autonomous games contributes to positive effects on the mental health of participants. The findings of Hirayanagi et al. [28] may be useful in developing perspective strategies against physical and mental fatigue associated with prolonged HDBR, horizontal bed rest, and microgravity environments. The study of Benvenuti et al. [29] showed that the observed ability of HDBR to inhibit cortical emotional responses raises an important issue on the risks that astronauts underestimate a dangerous/threatening situation or that long-term bedridden patients develop depressive symptoms.

Our team found different patterns of emotional changes under HDBR (short-duration and long-duration). Under short-duration HDBR (15d −6°), we used the BAI, BDI and PANAS as measurements. We found that HDBR caused female participants to undergo a transition from stressed to calm and from suffering to adaptive, along with changes in other physical symptoms [30]. Under long-duration HDBR (45d −6°), we used the BAI, BDI and PANAS with physiological indices (GSR and HR) and a neural index (frontal EEG asymmetry that represents emotion regulation competence). The results showed that when compared to the pre-HDBR period, the participants’ GSR and positive affect decreased significantly during the HDBR period [10]. That study suggests that an individual’s emotional fear before HDBR and emotional fluctuations in anxiety can be elevated in the early stage of HDBR, in which the simulated weightless environment induced somatic anxiety but not psychological anxiety in participants [31]. Under long-duration HDBR, people who preferred to maintain the stability of their emotional state made great effort to regulate their negative emotions. The association of frontal EEG asymmetry and positive affect may reflect the immediate ability to regulate emotion for astronauts during spaceflights [13]. The above findings are the results reported by our team, who focused on the effects of HDBR on human emotion.

To evaluate the effects of social isolation and sleep deprivation on an individual’s emotional state, we conducted an experiment in which participants underwent 72 h of sleep deprivation in social isolation. The profile of mood state (POMS) and PANAS were used as measurements. We found that when compared to social isolation, the experimental group under sleep deprivation was more likely to have negative moods, such as fatigue and panic, as well as negative emotions, such as irritation [9]. For real spaceflight (13 d), we used the PANAS and frontal EEG asymmetry as measurements. Our findings showed that because of well-practiced psychological countermeasures, astronauts maintained a high level of positive affect and did not suffer from the asthenia symptoms during short-duration spaceflight [14].

## Perspectives

Accordingly, a positive affect was sensible in the extreme environment, which showed that regardless of HDBR, sleep deprivation or spaceflight experiment compared to the pre-test (pre-flight), the scores of positive affect decreased after the experiment. First, the females’ positive affect remained at a stable level over the 15-day HDBR. However, at the end of the HDBR period and afterward, they demonstrated negative emotional states, such as decreased vigor and inappetence [32].

In addition, the effect of the 45-day HDBR on the males’ positive affect was that the negative affect remained relatively stable. However, the positive affect decreased at the end of HDBR and continued to decrease, which mainly included appetite and interest. This means the 45-day HDBR negatively affected the males’ positive affect. At the same time, this damage was in accordance with the GSR of males under HDBR, which demonstrated a decrease as soon as the participants began HDBR and remained at low levels during HDBR. After the HDBR period, the GSR went up and continued to increase after HDBR, but it did not return to the baseline. This was consistent with the ‘third-quarter phenomenon’ under the extreme environment, which suggests that an individual’s mood would have a tendency to fluctuate among prophase anxiety, metaphase boring and end-stage exciting.

Third, regardless of gender, males and females showed a very high level of positive affect under 13-day orbital spaceflight, which may relate to a short-duration time course and effective countermeasures used by the astronaut [14]. The neural EEG activity appeared to follow a similar trend for positive affect, suggesting a decreased ability to regulate emotion and increased aggravation of negative emotion vulnerability. We did not measure negative emotion because the participants’ EEG activity decreased after the spaceflight compared to the pre-flight, thus we deduced that the ability to regulate emotion may be decreased and the aggravation of negative emotion vulnerability may be increased according to the evidence presented in our previous study [13].

Finally, compared to the 72-hour social isolation condition, the participants who underwent 72 h of sleep deprivation (SD) showed more negative emotions, such as fatigue and panic, after SD, which indicates that SD increased the participants’ negative mood because of changes in circadian rhythms rather than the social isolation condition itself. Finally, stress influenced the sex and group moderator effect and specifically showed an effect on the emotional reactivity, emotional regulation and coping strategy for men and women. This reflects individual and group differences when facing stressors and gives us insight into crew member arrangement, mobilization and intervention.

## Conclusions

The effects of spaceflight and its analogs on individual (female and male astronauts) positive affect were moderated by the time duration. During the short-duration experiment (13-day orbital spaceflight and 15-day HDBR), the positive affect remained to be stable. While during the long-duration experiment (45-day HDBR), the positive affect decreased significantly at the end of the HDBR and after the HDBR. These indicated that more countermeasures of emotion regulation were necessary during long-duration spaceflight. These may give insights into the importance of emotion training for the success and safety for astronauts’ spaceflight mission.

## Abbreviations

ANS: Autonomic nervous system
BAI: Beck anxiety inventory
BDI: Beck depression inventory
EEG: Electroencephalogram
GSR: Galvanic skin response
HDBR: Head-down bed rest
HR: Heart rate
HRV: Heart rate variability
ISS: International space station
PANAS: Positive affect and negative affect scale
PNS: Parasympathetic nervous system
POMS: Profile of mood state
SD: Sleep deprivation
SNS: Sympathetic nervous system
